# Living with diabetes in the Brazilian Amazon: Exploring barriers and supports for healthy lifestyle practices

**DOI:** 10.1111/dme.70381

**Published:** 2026-07-15

**Authors:** Thalyta Mariany Rêgo Lopes Ueno, Tiago Assunção dos Santos Farias, Wagner Ferreira Monteiro, Maria Paula dos Santos, Hércules Lázaro Morais Campos, Elisa Brosina de Leon

**Affiliations:** ^1^ Programa de Pós‐graduação em Enfermagem em Saúde Pública, Escola de Ciências da Saúde Universidade Do Estado Do Amazonas (UEA) Manaus Amazonas Brazil; ^2^ Programa de Pós‐graduação em Ciências Do Movimento Humano (PPGCiMH), Faculdade de Educação Física e Fisioterapia (FEFF) Universidade Federal do Amazonas (UFAM) Manaus Amazonas Brazil; ^3^ Faculdade de Desporto – Universidade do Porto (FADEUP) Porto Portugal; ^4^ Instituto de Saúde e Biotecnologia (ISB) Universidade Federal do Amazonas (UFAM) Coari Amazonas Brazil; ^5^ Programa de Pós‐graduação Interdisciplinar em Estudos Rurais Universidade Federal dos Vales do Jequitinhonha e Mucuri (UFVJM) Diamantina Minas Gerais Brazil

**Keywords:** barriers, contextual factors, facilitators, primary health care, public health, qualitative research, type 2 diabetes mellitus

## Abstract

**Aims:**

To investigate the barriers and facilitators to adopting healthy lifestyle habits among patients with type 2 diabetes mellitus (T2DM) in the Brazilian Amazon, using the Environmental Factors domain of the International Classification of Functioning, Disability and Health (ICF) as a conceptual framework.

**Methods:**

A qualitative study was conducted with 47 patients with T2DM and 17 community health workers (CHWs) from primary health care services in the Brazilian Amazon. Data were collected through two World Café group discussion sessions (one involving patients and one involving CHWs) and 15 semi‐structured interviews conducted in patients’ homes. Data were analysed using thematic analysis supported by Atlas.ti 24 software.

**Results:**

Three major categories emerged: attitudinal, social and physical factors. Attitudinal barriers included resistance to behaviour change, denial of disease severity and physical limitations, whereas fear of complications and valuing life motivated healthier behaviours. Social barriers comprised lack of family support, insecurity and transportation difficulties, while family involvement, professional guidance and trust in CHWs facilitated treatment adherence and lifestyle modification. Physical barriers included inadequate infrastructure, adverse weather conditions and the absence of safe public spaces for physical activity. Conversely, proximity to local markets and fairs facilitated access to healthy foods and supported dietary improvements.

**Conclusions:**

Barriers and facilitators to healthy lifestyle adoption among people with T2DM in the Brazilian Amazon are strongly influenced by attitudinal, social and environmental factors. Effective diabetes management in this context requires culturally sensitive interventions, improved urban infrastructure, strengthened community support networks and continued engagement of multidisciplinary healthcare teams, particularly CHWs. These findings provide important insights for developing context‐specific strategies to promote sustainable lifestyle changes, enhance patient autonomy and improve quality of life in remote Amazonian communities.

**What's new?**

- Environmental, social, and attitudinal factors strongly influence adherence to healthy lifestyle behaviours among adults with type 2 diabetes living in the Brazilian Amazon.
- Family support, trusted community health workers, and access to local healthy foods facilitate diabetes self‐management, whereas poor infrastructure, geographic isolation, and insecurity act as major barriers.
- Diabetes interventions in remote Amazonian settings should incorporate culturally tailored strategies and address contextual barriers beyond individual behaviour change.

## 1 INTRODUCTION

Living in the remote Brazilian Amazon imposes unique complexities on type 2 diabetes mellitus (T2DM) management, where social vulnerability, cultural diversity, geographic isolation and limited health service access compound the disease burden.^1^, ^2^ Although T2DM is a prevalent condition heavily influenced by lifestyle behaviours,^3^, ^4^ these habits are constrained by barriers to access to information and specialized care.^3^ Despite initiatives to reinforce prevention in primary care,^5^ progress in non‐pharmacological management, particularly regarding dietary practices and physical activity, remains stagnant.^4^, ^5^, ^6^ Compounding these structural limitations, a notable lack of intrinsic motivation and low health awareness further hinder treatment compliance,^7^ illustrating the tight interplay between personal and environmental barriers. In contrast, CHWs emerge as vital facilitators within the community, playing an indispensable role in supporting healthier lifestyles.^8^

Although public policies aimed at preventing and managing T2DM exist, such as actions under the Family Health Strategy, they do not always adequately address the complexities of the territories and patients' lives.^9^ Adherence to healthy habits, such as a balanced diet and physical activity, is hindered by structural factors like the absence of public transportation, food insecurity and the lack of appropriate spaces for exercise.^10^ Improving the physical and social environment and investing in health education are key strategies for encouraging self‐care practices and promoting T2DM control.^11^ Beyond environmental dimensions, individual components play a crucial role in treatment adherence. Elements such as intrinsic motivation, health literacy, emotional resilience and personal beliefs about the severity of the illness directly influence how the patient interacts with their surrounding environment.^12^

The study uses the International Classification of Functioning, Disability, and Health (ICF) as a theoretical framework,^13^ given its potential to integrate biological, social and environmental health components. The ICF enables analysis beyond the disease, considering the contexts and ways of life that directly affect functionality and social participation. The investigation is based on the hermeneutic‐phenomenological tradition, drawing on the philosophical principles of Gadamer and Ricoeur^14^, ^15^ to interpret participants' lived experiences and the subjective meanings they attribute to their health routines. This approach is particularly necessary because existing research on adherence to treatment for T2DM is predominantly conducted in high‐income Western countries, producing results that cannot be generalized to diverse cultural and socioeconomic realities, as is the case in the Brazilian Amazon.^16^

This study aims to understand the barriers and facilitators to adopting healthy lifestyle habits among patients with T2DM from the perspective of the ICF's contextual factors within primary healthcare in the interior of Amazonas.

## 2 METHODS

A qualitative study with a participatory interface. Participatory research involves healthcare professionals, students, patients and community members as co‐researchers, promoting the co‐production of knowledge and greater relevance of findings.^17^ The study is part of a larger project titled ‘Intervention Led by Community Health Workers for the Management of Type 2 Diabetes in the Interior of Amazonas’. The research used the Consolidated Criteria for Reporting Qualitative Research (COREQ) checklist.^18^ Data collection took place between August and October 2023.

This study also draws on the hermeneutic‐phenomenological perspective, as developed by Gadamer and Ricoeur,^14^, ^15^ understanding that the meaning of participants' lived experiences is constructed through language, historical‐social context and interactions. Data analysis sought to go beyond thematic description, interpreting the meanings attributed by patients and Community Health Workers (CHWs) to the barriers and facilitators to adopting healthy habits, in light of their social and cultural realities. This theoretical orientation allowed for the valuation of subjectivities, emerging meanings and the circularity of understanding, central elements of a qualitative analysis sensitive to the Amazonian context.

The study involved 17 CHWs and 47 patients with T2DM, all from six Primary Health Care (PHC) Units in the city of Iranduba, Amazonas, Brazil. CHWs affiliated with PHC and patients with T2DM aged over 18 years, with at least 6 months of engagement with one of the PHCs, regardless of sex or race, were included. CHWs on leave, vacation or absenteeism, and patients with T2DM presenting physical, intellectual or communicative limitations that could impair participation were excluded.

Data collection was carried out using the World Café technique.^19^ Meetings were organized at different times: mornings for patients and afternoons for CHW. Each meeting was structured in two phases, called ‘Prosa e Café’. The first phase discussed barriers to adopting healthy eating habits, and the second identified facilitators of these changes.

The research team received training in monitoring dynamics and facilitating group discussions, and during the World Café, participants completed a sociodemographic questionnaire. The instrument collected sociodemographic data for patients regarding age (years), profession, gender, household income, level of schooling, monthly family income, time elapsed since diagnosis of type 2 diabetes and use of medications for treatment; for CHWs, data were collected regarding age (years), length of experience as a CHW, gender and level of schooling.

The World Café sessions began with an introductory presentation led by the study coordinators. This initial step aimed to establish trust, outline the study objectives and explain the World Café dynamics, including the logistics of table rotation and the role of the hosts. Instead of using structured, rigid question scripts, collaborative dialogue was stimulated by specific thematic axes assigned to each table, which served as starting points. Depending on the phase of the session, discussions focused strictly on environmental barriers or facilitators related to healthy eating habits, allowing participants to freely share their daily experiences and contextual challenges within that specific dimension. Excerpts from participants' statements during the World Café were identified using codes: ‘US’ for patients (US1–US32) and ‘A’ for CHWs (A1–A17) to ensure confidentiality and anonymity.

Semi‐structured interviews were conducted with T2DM patients who did not participate in the sessions to deepen the data obtained in the World Café. From a list provided by the CHW, 15 of 25 patients were interviewed at their homes. Interviews were recorded and stored on Google Drive, with patients identified by codes U1–U15. Data collection followed the theoretical saturation criterion and was interrupted when no new perceptions or relevant themes emerged.^20^

The home interviews were conducted using a semi‐structured guide designed to capture the participants' lived experiences through a phenomenological lens. The interview dialogue began with broad, open‐ended questions that explored the participants' conceptions of a healthy lifestyle and how they believed it could be achieved. Subsequently, the dialogue moved into specific environmental dimensions, guided by the following central triggering question: ‘In your conception, which barriers and facilitators regarding environmental factors favour or hinder your adherence to healthy habits?’ This progression allowed participants to first define their health perspectives before detailing the specific physical, social and attitudinal challenges within their territory.

Data analysis was conducted using the Atlas. ti 24 software (Qualitative Research and Solutions)^21^ and followed the guidelines of Thematic Network Analysis,^22^ aligned with the adopted conceptual framework. Initially, the research team developed a set of deductive codes, which were refined through a second coding round. In this phase, patterns and themes emerged, forming the Thematic Network titled ‘Barriers and Facilitators to Adopting Healthy Lifestyle Habits among Amazonian Patients with Type 2 Diabetes Mellitus’. Within the main themes, 11 codes related to barriers and 18 related to facilitators were identified.

The study was conducted in accordance with the ethical principles established by Resolutions Nos. 466/2012 and No. 510/2016 of the Brazilian National Health Council. The Research Ethics Committee (CEP) of the Federal University of Amazonas (UFAM) approved the project under opinion No. 5.931.419, and the Municipal Health Secretariat of Iranduba authorized it. All participants signed the Informed Consent Form and authorized the use of their images and voices.

## 3 RESULTS

Most participants were female, in both the CHW and patient groups, totalling 81.25% (52), with ages ranging from 30 to 79 years. Among the patients, the most common education level was ‘Incomplete Primary Education’, corresponding to 71.88% (23), while among the CHW, ‘Completed Secondary Education’ predominated, representing 52.94% (9) (Figure 1).

**FIGURE 1:**
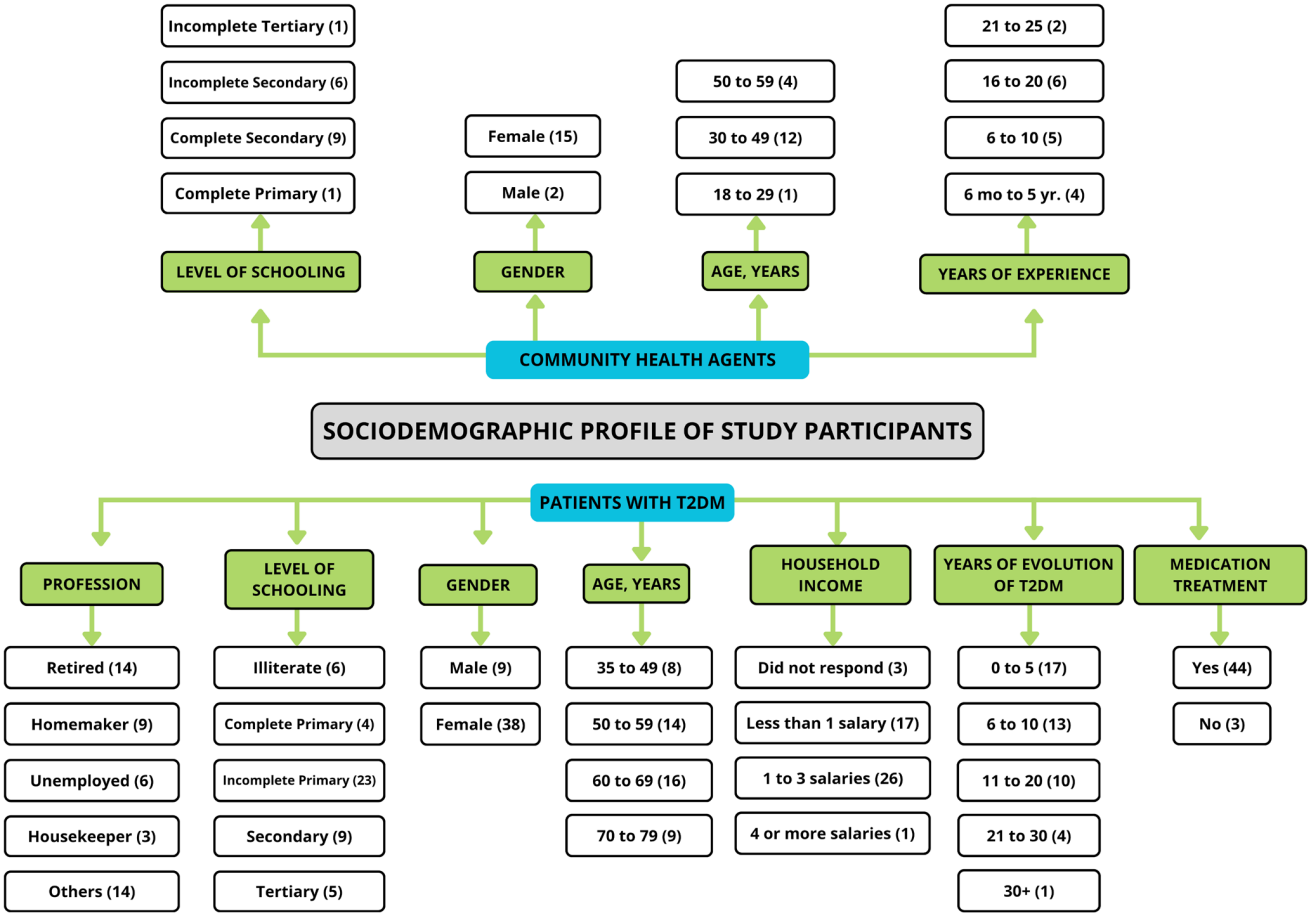
Characterization of the research participants.

The thematic network ‘Environmental Barriers and Facilitators for the Adoption of Healthy Lifestyles in Amazonian Patients with Type 2 Diabetes Mellitus’ organized the themes into three main categories: attitudinal, social and physical factors. These topics are summarized in Figure 2 and aligned with the ICF, highlighting the impacts of environmental and personal factors on people with T2DM.^23^

**FIGURE 2:**
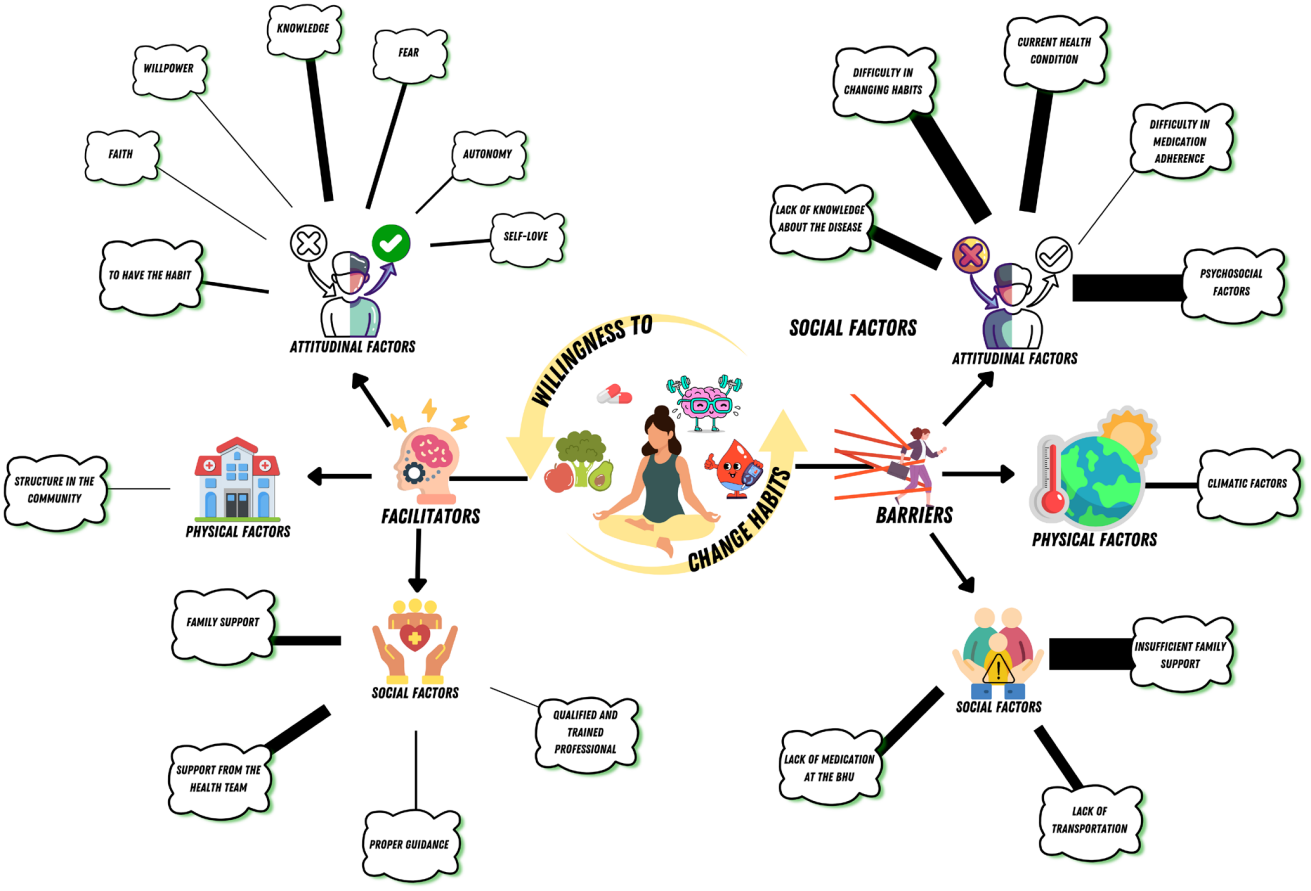
‘Prosa e Café’ thematic network results.

### 3.1 Attitudinal factors

Attitudinal factors: the organizational theme that gathers patients' perceptions and attitudes that act as obstacles to lifestyle changes. Highlights include attachment to previous routines, disbelief in the severity of T2DM and physical limitations that reduce motivation for healthy practices.

U6: ‘Adaptation. I have to adapt, and adapting is difficult because you already have a life routine. […] Changing is difficult!’

A2: ‘She said that either way, she would die. So, in other words, for her, the pleasure of eating is greater than her own life.’

US7: ‘I feel pain, leg pain, difficulty seeing, all of which make it hard to exercise […]’

These findings reveal that health‐related decisions are not limited to biomedical knowledge but are also intertwined with beliefs, emotions and perceptions of identity, requiring more dialogical and culturally sensitive educational approaches.

### 3.2 Social factors

The social factors theme includes aspects such as a lack of family support, insecurity and mobility difficulties that hinder adherence to healthy practices.

US1: ‘I lack help from my family; they do not let me follow a diet. I cook for everyone at home and do not think about myself.’

U4: ‘Lack of security, the area is dangerous. What we see a lot is robbery.’

A3: ‘Difficulty getting to the health unit, no money for transportation. Imagine using transportation to exercise.’

Social factors accounts show how support networks and urban environments directly interfere with patients' ability to make choices and change behaviours.

### 3.3 Physical factors

Local environmental conditions, such as intense heat, rain and inadequate infrastructure, emerge as concrete barriers to physical activity.

US5: ‘We do not have any proper place to walk or exercise.’

US8: ‘We walk on the curb, dodging cars, motorcycles, at risk of being hit.’

A10: ‘The heat is scorching, dusty, and now smoke. […] When it is not that, it is rain and mud.’

Ambient conditions point to the need for public policies that integrate health and urban planning, especially in regions like the Amazon.

### 3.4 Attitudinal factors

Attitudinal factors: individual attitudes that encourage change, such as fear of disease complications, appreciation of life and engagement in pleasurable activities that promote well‐being.

U10: ‘Fear of dying, fear of leaving my daughters.’

A4: ‘She stopped eating flour because she understood it was harmful.’

US9: ‘Realizing that life is important, that being alive is good. That is a motivation.’

A3: ‘Playing guitar, sewing, taking care of plants. […] These are facilitators to rethink life and change.’

The statements reinforce that health care is deeply connected to subjective and emotional meanings, which must be considered in educational processes.

### 3.5 Social factors

Family and community support, as well as strong bonds with health professionals, appear to be significant drivers of healthy habit adoption.

US2: ‘Family support […] also helped me change.’

U13: ‘Medical guidance helped me change my behaviors.’

US7: ‘My community health agent is very good; she comes to my house whenever I need […] and responds at any time.’

The interactions between family and health service demonstrate that health care goes beyond technical aspects: it involves trust, listening and bonds between professionals and the community.

### 3.6 Physical factors

Physical factors refer to environmental and structural conditions that favour healthy practices, such as nearby markets, access to fresh foods and community transportation.

U1: ‘If it were in a plaza, this transport would greatly help.’

A8: ‘A project with a nutritionist, transportation, and a place for activities should be created.’

US9: ‘The small nearby market stalls selling fish, vegetables, and herbs […]’

The accounts highlight the central role of territory in promoting or inhibiting health, reinforcing the importance of intersectoral collaboration. The integration of data from World Café sessions and household interviews revealed a strong convergence regarding barriers to lifestyle changes. While the dynamics of the World Café brought to light a collective consensus in which both CHWs and patients emphasized institutional neglect and structural limitations of the territory as the main obstacles, individually, the consensus built during group interactions translated into a shared feeling of resignation among patients, who perceive environmental barriers not only as daily inconveniences but as absolute impediments that shape their health choices and distance them from what they conceive as a healthy life.

## 4 DISCUSSION

This study reveals how contextual factors shape the management of type 2 diabetes in the Brazilian Amazon. Based on the ICF framework, our results show that environmental factors, such as geographic isolation, complex river transport and structural gaps in health services, represent significant barriers to lifestyle modification. On the other hand, cultural identity and the presence of CHW act as vital facilitators. This environmental reality directly intersects with personal factors, in which low health literacy and diminished motivation often stem from these territorial vulnerabilities rather than individual failures, ultimately dictating adherence to dietary and physical activity habits.^24^

The sociodemographic profile of the participants shows a predominance of females (81.25%) and ages ranging from 30 to 79 years, aligning with studies indicating a higher prevalence of type 2 diabetes among women and older age groups.^23^ Additionally, the finding that patients mostly had incomplete elementary education and CHWs had completed high school reflects the educational limitations characteristic of the Northern region,^25^ resulting in a higher risk of complications associated with T2DM. This scenario highlights the urgent need for educational interventions to promote understanding and proper management of T2DM.^26^

The analysis of the results, anchored in the ICF,^13^ provides a broader understanding of the barriers and facilitators to adopting healthy habits among patients with T2DM in the Amazon region, particularly regarding the attitudinal, social and physical factors that shape their daily practices. From a hermeneutic‐phenomenological perspective, the meanings attributed to self‐care emerge from the participants' unique experiences and cultural context, reflecting the ‘fusion of horizons’ and the ‘possible worlds of meaning’ proposed by Gadamer and Ricoeur.^14^, ^15^ These findings show that health care is not limited to the application of technical procedures but is also immersed in historically constructed values, emotions and understandings.

The thematic network proposed in this study highlighted the complexity of interactions among these factors in promoting health among individuals with diabetes, a context further deepened by the identification of three main themes: attitudinal, social and physical factors.

### 4.1 Attitudinal factors

The results indicate that attitudinal factors, such as difficulty adopting new habits and disbelief in the severity of the disease, are significant barriers to adherence to healthy practices.^27^ A participant's statement, expressing the idea ‘either way, I am going to die’, reveals resistance to change and denial of the disease's severity, which may lead to minimization of care and impaired treatment adherence.^28^

Additionally, pain and physical limitations reported by some participants, such as difficulty seeing and exercising, suggest that the disease's direct impact on functionality also interferes with the motivation to change.^29^ Other studies have also observed that physical condition and other comorbidities can significantly hinder engagement in physical activities.^30^

However, attitudinal factors that facilitate the adoption of healthy habits were also identified. Fear of serious complications and/or perceived threats, such as the risk of a stroke, emerged as important motivations for decision‐making.^31^ Another facilitating factor was the perceived harm caused by certain foods.^32^

Motivation for change was also observed in aspects related to self‐esteem and the value of life.^33^ Additionally, engaging in hobbies was mentioned as emotional support, promoting reflection on life and facilitating behavioural changes.^34^

### 4.2 Social factors

The analysis of social factors revealed that a lack of family support, social insecurity and transportation difficulties are barriers to adopting healthy habits.^35^ Furthermore, neighbourhood insecurity and financial difficulties in accessing health units and engaging in physical activity corroborate studies indicating a strong correlation between socioeconomic conditions and treatment adherence.^36^

Social, family^37^ and professional^38^ support are essential for adherence to healthy habits among patients with T2DM. Testimonies, such as that of US2, highlight how family involvement facilitates the adaptation to a new routine of eating and self‐care, strengthening patients' motivation.^39^ Guidance from health professionals, as reported by U13, is also crucial to help patients understand the importance of proper treatment.^40^ Another important factor is the continuous support from the multiprofessional team, evidenced by US7, who highlighted the availability of the CHW.^41^

This personalized follow‐up, combined with trust in local figures and agile service, facilitates treatment adherence, especially in regions like the Amazon, where access to health services is limited.^42^ The CHW management reinforces the need for an integrated health system with qualified professionals and strong family support.^43^

### 4.3 Physical factors

Environmental conditions and poor urban infrastructure strongly influence physical factors. Participants mentioned the lack of adequate exercise spaces, which exemplifies the physical barriers to maintaining an active lifestyle.^44^

The Amazon region's climatic conditions also pose specific challenges, including high temperatures, heavy rainfall and muddy terrain, which hinder outdoor physical activities.^44^ The perception that the environment is unfavourable for exercise reflects the region's geographical and climatic challenges, which necessitate specific interventions.

Proximity to markets and fairs facilitates access to healthy foods, which are essential for T2DM control. These findings reinforce the importance of environments that support healthy food choices and regular physical activity, particularly in overcoming local infrastructure and transportation limitations.^45^

One of the main strengths of this study is its investigation in a little‐studied Amazonian region, combining the perspectives of patients and CHW through thematic networks. However, some limitations should be considered, particularly the purposive sampling strategy, which prevents statistical generalization or measurement of incidence. These insights reflect the specific context of this territory and, while offering valuable applicability to other isolated and resource‐limited communities, caution is warranted when applying them to urbanized healthcare settings.

## 5 CONCLUSION

This study highlighted the complexity of attitudinal, social and physical factors influencing the adoption of healthy habits among patients with T2DM in the Amazon region. Barriers such as a lack of family support, inadequate infrastructure and adverse climatic conditions hinder the adoption of healthy practices. In contrast, factors like family support, professional guidance and proximity to markets and fairs were identified as key facilitators.

The structural conditions in the Amazon call for local and regional public policies aligned with international recommendations for promoting healthy environments. Additionally, the continuous involvement of the multi‐professional team, especially the CHW, proved fundamental in providing emotional and educational support for habit change among patients.

Despite the consistent results, this study has limitations due to its conduct in a single municipality in the Amazon, which may limit the generalizability of the findings. Strengthening local infrastructure, expanding community support and developing culturally sensitive educational strategies are recommended to promote healthy habits and improve adherence to T2DM treatment. These actions can enhance patients' autonomy and positively impact their quality of life.

Based on these findings, future research should focus on implementation studies for the joint development of health interventions that respect the unique geographic and cultural realities of remote Amazonian communities. Concomitantly, longitudinal and mixed‐methods studies are needed to assess the long‐term impact of CHW‐led strategies on patient motivation and health knowledge. Expanding this framework to other vulnerable and resource‐limited territories will be essential for testing the transferability of these contextual indicators and supporting the creation of more equitable chronic disease policies in primary care.

## FUNDING INFORMATION

Fundação de Amparo à Pesquisa do Estado do Amazonas (FAPEAM–FAP/CnPQ003/2022), Conselho Nacional de Desenvolvimento Científico e Tecnológico (CnPQ) and Coordenação de Aperfeiçoamento de Pessoal de Nível Superior (CAPES – 001) supported the research.

## CONFLICT OF INTEREST STATEMENT

The authors declare no conflict of interest.

## ETHICS STATEMENT

The study was conducted in accordance with the ethical principles established by Resolutions Nos. 466/2012 and No. 510/2016 of the Brazilian National Health Council. The Research Ethics Committee (CEP) of the Federal University of Amazonas (UFAM) approved the project under opinion No. 5.931.419 and the Municipal Health Secretariat of Iranduba authorized it. All participants signed the Informed Consent Form and were authorized to use their images and voices.
